# Clinical diagnosis and treatment of transdiaphragmatic intercostal hernia: a retrospective study based on 40 cases

**DOI:** 10.3389/fsurg.2025.1674085

**Published:** 2025-10-29

**Authors:** Wei Wu, Tianzhu Zhang, Yiguo Zhao, Xiaodong Xun, Pengji Gao

**Affiliations:** 1 Department of General Surgery, Beijing Jishuitan Hospital, Capital Medical University, Beijing, China; 2 Department of Emergency Surgery, General Hospital of Xining First Medical Group, Xining, China

**Keywords:** diaphragm, intercostal hernia, trauma, obesity, transdiaphragmatic intercostal hernia

## Abstract

**Objective:**

To retrospectively analyze the clinical characteristics, diagnostic methods, and treatment strategies of transdiaphragmatic intercostal hernia (TDIH), so as to provide systematic clinical evidence for the diagnosis and treatment of TDIH.

**Methods:**

The data of 1 patient with TDIH diagnosed in our hospital in 2024 was reviewed. Relevant case literatures were retrieved from the Pubmed database (from 1968 to 2024), CNKI, and Wanfang Data Platform (as of November 1st, 2024). Clinical data such as age, gender, predisposing factors of onset, clinical symptoms, surgical methods, and complications were collected and sorted out. Graphpad Prism9.5.1 was used for statistical analysis and graphing.

**Results:**

A total of 40 cases of TDIH clinical data were included. The average age of the patients was 62.5 ± 13.7 years, with 30 male patients (75%) and 10 female patients (25%). Trauma was the main predisposing factor. Common clinical symptoms included dyspnea, abdominal pain, etc. The hernia sac was mostly located in the left intercostal space. Surgery was the main treatment method. 75% of the patients underwent open surgery, and 15% of the patients received minimally invasive surgery. Common hernia contents included colon, small intestine, omentum, etc. 50% of the patients had a mesh placed during the operation. 88% of the patients had no obvious postoperative complications, while 12% had complications such as pneumothorax.

**Conclusion:**

TDIH is rare and prone to misdiagnosis and missed diagnosis. CT scanning is a crucial diagnostic means. Surgery is the main treatment method. Postoperative management is important. Risk factor analysis identified obesity (aHR 2.05, 95% CI 1.18–3.56) and large defect size (>5 cm; aHR 2.41, 95% CI 1.39–4.18) as independent risk factors for postoperative adverse events. In the future, more accurate diagnostic methods and individualized treatment regimens need to be explored to improve the prognosis.

## 1 Introduction

Transdiaphragmatic intercostal hernia (TDIH) is a rare condition typically caused by traumatic diaphragmatic injury, leading to the protrusion of abdominal contents through a diaphragmatic defect and into the thoracic cavity along the intercostal space or incarceration within the intercostal region (1, 2). First documented in the late 1970s, TDIH has been increasingly recognized with advances in cross-sectional imaging. Historically, diagnosis was often delayed or missed due to nonspecific symptoms and limited imaging capabilities. The evolution of computed tomography (CT) has revolutionized preoperative planning, enabling precise localization of diaphragmatic injuries and herniated viscera. Although the incidence of TDIH is low (3), early diagnosis remains challenging due to its frequently insidious clinical presentation and susceptibility to misdiagnosis. TDIH is commonly associated with a history of trauma, particularly blunt or penetrating trauma, and symptoms may be mild, often requiring detailed medical history and imaging examinations for definitive diagnosis (3, 4). Computed tomography (CT) is the gold standard for diagnosing TDIH (4), as it helps localize the diaphragmatic injury and identify the herniated contents. Surgical repair is the primary treatment, including open surgery, laparoscopic surgery, and robot-assisted techniques (5). The surgical approach has diversified over time, from primarily open repairs to the increasing adoption of minimally invasive techniques, which offer potential benefits of reduced morbidity and faster recovery. In recent years, minimally invasive surgery has been widely adopted due to its reduced invasiveness; however, postoperative recurrence and complications, such as adhesions and mesh rejection, remain significant challenges in clinical management (5). While previous case reports and small series have documented TDIH, a comprehensive synthesis of its clinical spectrum and evidence-based analysis of risk factors for poor outcomes is lacking in the literature. This study aims to fill this knowledge gap by. This study reviews a TDIH case treated in our hospital and analyzes 39 additional cases from the literature to summarize the clinical characteristics, diagnostic approaches, and treatment strategies, aiming to provide a more systematic clinical reference for the diagnosis and management of TDIH.

## 2 Methods

### 2.1 Clinical data

We retrospectively reviewed a case of transdiaphragmatic intercostal hernia (TDIH) managed at our institution in 2024. A systematic literature search was performed in PubMed (1968–2024) using the terms “transdiaphragmatic intercostal hernia” and “intercostal pleuroperitoneal hernia.” Chinese databases (CNKI, Wanfang Data) were also queried (up to November 1, 2024) with the keywords “transdiaphragmatic intercostal hernia,” “intercostal pleuroperitoneal hernia,” and “intercostal hernia.” The reference lists of retrieved articles were manually screened for additional relevant cases. Included studies met the following criteria: Confirmed TDIH diagnosis, Full-text availability in English or Chinese, Non-duplicate case reports. Excluded: Non-English/Chinese articles, redundant publications.

### 2.2 Data extraction

Data extraction covered: age, sex, trauma history, predisposing factors, symptoms, hernia location; obesity status (defined as BMI ≥30 kg/m^2^or explicitly described as “obese” in the original report), surgical approach (open/laparoscopic/robotic), mesh use, mesh type (absorbable or non-absorbable, when available), herniated organs, postoperative complications. For missing data, cases were excluded from specific analyses where that variable was required.

### 2.3 Risk factor analysis

Potential prognostic factors were evaluated using: Univariate Cox regression for time-to-event outcomes (survival/recurrence). Multivariate analysis with backward stepwise selection (*p* < 0.1 for entry). Adjusted for clinically relevant confounders. Sensitivity analysis excluding cases with missing data. Cox regression was chosen for its ability to handle time-to-event data and censoring, which is appropriate for analyzing outcomes like recurrence and complications even with varying follow-up periods across studies.

### 2.4 Statistical analysis

Data were analyzed using Microsoft Excel 2019 and visualized with GraphPad Prism 9.5.1. Continuous variables are expressed as mean ± SD; categorical variables as percentages. Statistical significance was set at two-tailed *p* < 0.05. Hazard ratios (HRs) with 95% confidence intervals (CIs) were reported.

## 3 Results

### 3.1 Case report from our institution

A 50-year-old male patient was admitted with an 8-month history of a reducible left intercostal mass. Eight months prior, the patient had been involved in a motor vehicle accident and was diagnosed with multiple left rib fractures, traumatic wet lung, and pneumothorax at a local hospital. After conservative treatment and discharge, he noticed a reducible left intercostal mass but did not seek treatment. The mass gradually enlarged with accompanying pain, prompting his visit to our hospital.

**Figure 1:**
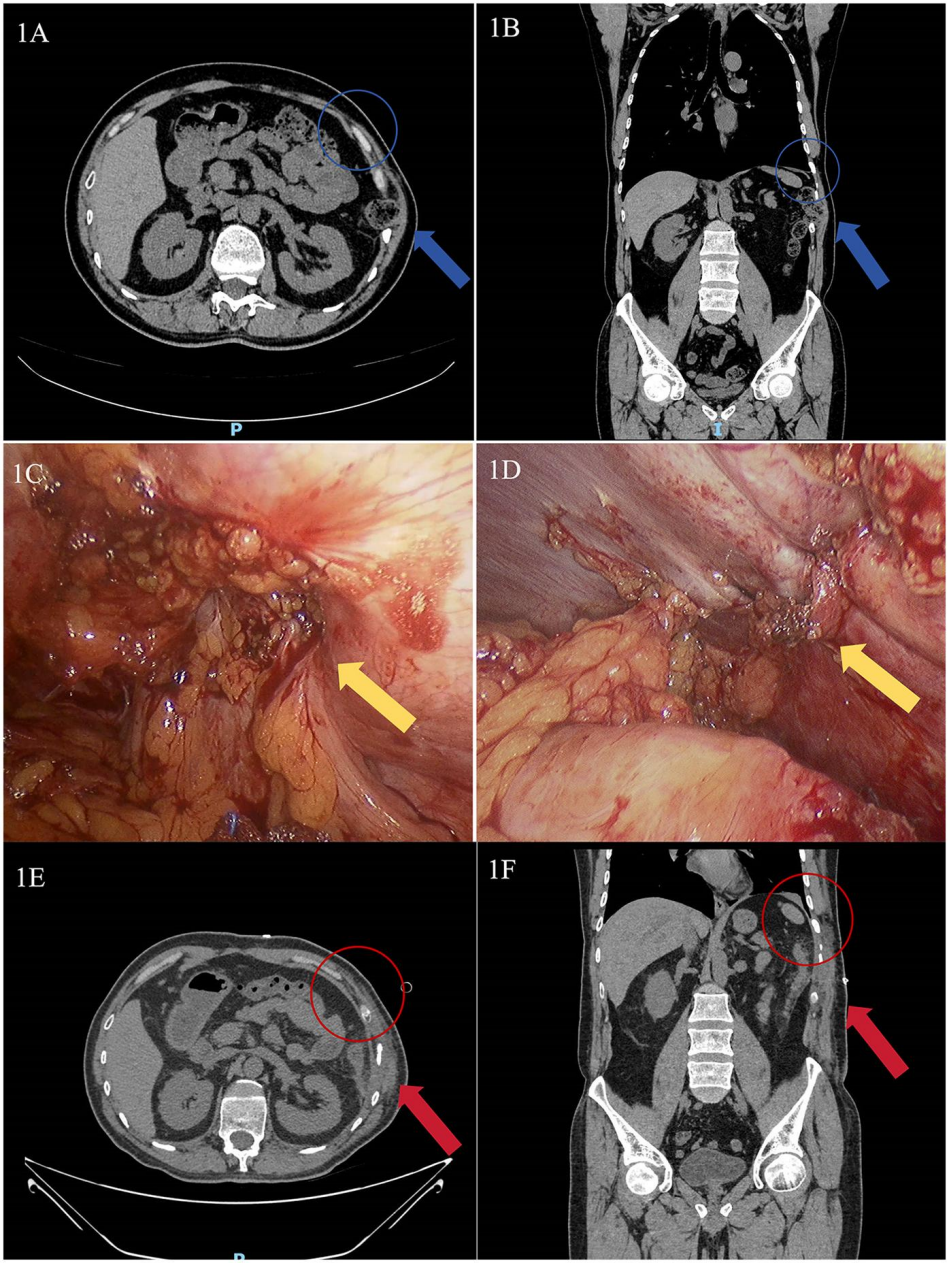
Abdominal CT and intraoperative images of the patient treated in our hospital. **(A)** Preoperative axial abdominal CT image (The blue arrow indicates the location of the intercostal hernia, the blue circle demarcates the area of diaphragmatic rupture); **(B)** Preoperative coronal abdominal CT image (The blue arrow indicates the location of the intercostal hernia, the blue circle demarcates the area of diaphragmatic rupture); **(C)** Intraoperative view of the hernia sac (The yellow arrow indicates the site of defect, with the colon herniated through it); **(D)** Intraoperative view after diaphragmatic defect repair (The yellow arrow denotes the post-repair site. The defect was closed with interrupted sutures, and no mesh was deployed); **(E)** Postoperative axial abdominal CT image (The red arrow indicates the successfully repaired defect with resolution of the hernial sac; the red circle demonstrates the restored continuity of the diaphragm); **(F)** Postoperative coronal abdominal CT image (The red arrow indicates the successfully repaired defect with resolution of the hernial sac; the red circle demonstrates the restored continuity of the diaphragm).

Physical examination revealed a 7 × 8 cm soft, tender mass without fluctuance in the left 10th-11th intercostal spaces along the anterior axillary and midaxillary lines. The skin appeared normal without erythema or ulceration. The mass exhibited mild respiratory movement, with a palpable intercostal weak area showing notable impulse during coughing. Abdominal CT demonstrated a left 10th-11th intercostal abdominal wall hernia (Figures 1A,B).

The diagnosis of transdiaphragmatic intercostal hernia (TDIH) was confirmed, and the patient underwent laparoscopic intercostal hernia repair under general anesthesia. The patient was placed in a supine position. A standard three-port laparoscopic configuration was used. Intraoperative findings revealed a diaphragmatic defect at the left 10th-11th intercostal space with herniation of the descending colon (Figure 1C). The incarcerated colon was reduced, and the diaphragmatic defect was repaired with interrupted 2-0 Prolene sutures (Figure 1D). A synthetic mesh was not used as the defect was deemed repairable under minimal tension. The patient recovered well and was discharged on postoperative day 7. One-month follow-up CT showed no abnormalities (Figures 1E,F), with no recurrence observed during 6 months of follow-up.

### 3.2 Clinical characteristics of TDIH

**Figure 2:**
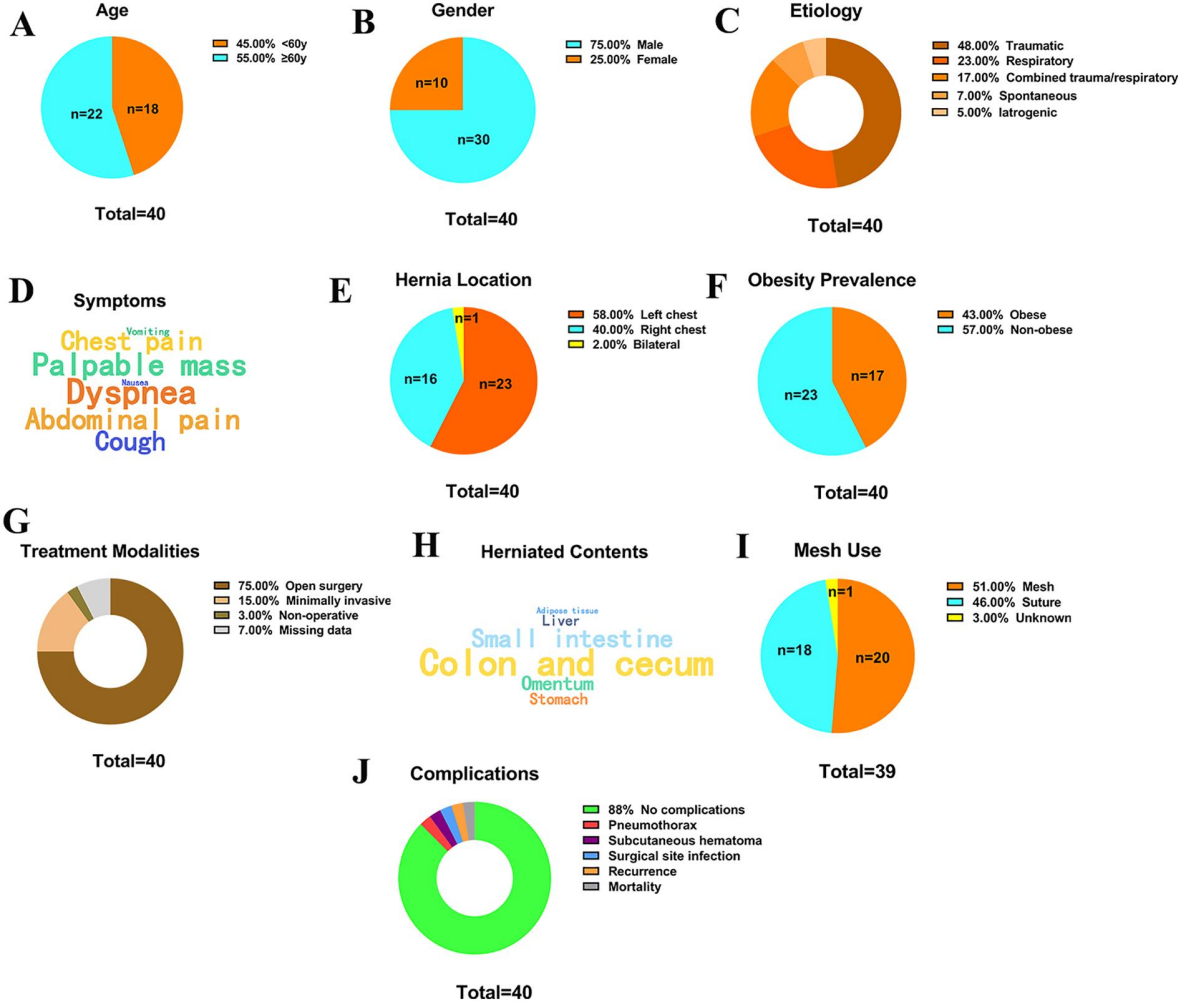
Clinical characteristics of patients with transdiaphragmatic intercostal hernia. **(A)** Age distribution; **(B)** Gender distribution; **(C)** Predisposing factors; **(D)** Word cloud of clinical symptoms (larger font indicates higher frequency); **(E)** Location of the hernia sac; **(F)** Proportion of obese patients; **(G)** Treatment methods; **(H)** Word cloud of hernia contents (larger font indicates higher frequency); **(I)** Mesh placement; **(J)** Complications.

Our study included 40 TDIH cases (1 from our institution and 39 from literature (1–37). The mean age was 62.5 ± 13.7 years, with 22 patients (55%) aged ≥60 years and 18 (45%) < 60 years (Figure 2A). There were 30 males (75%) and 10 females (25%) (Figure 2B). Etiological analysis showed:Traumatic causes: 19 cases (48%), Respiratory diseases: 9 cases (23%), Combined trauma/respiratory: 7 cases (17%), Spontaneous: 3 cases (7%), Iatrogenic: 2 cases (5%) (Figure 2C). Common symptoms included dyspnea, abdominal pain, chest pain, cough, and palpable mass (Figure 2D). Other manifestations included swelling, nausea/vomiting, intestinal obstruction, and hematoma. Hernia locations were: Left-sided: 23 cases (58%), Right-sided: 16 cases (40%), Bilateral: 1 case (2%) (Figure 2E). Obese patients accounted for 43% (17/40) of cases (Figure 2F).

### 3.3 Treatment modalities

Surgical management was predominant: Open surgery: 30 cases (75%), Minimally invasive (laparoscopic/thoracoscopic/robotic): 6 cases (15%), Non-operative (adaptive servo-ventilation): 1 case (3%), Missing data: 3 cases (7%) (Figure 2G). Common herniated contents included: Colon/cecum, Small intestine, Omentum, Stomach, Liver, Lung, Adipose tissue (Figure 2H). Mesh was used in 20 cases (51%), while 18 (46%) underwent primary suture repair. Data were unavailable for 2 cases (3%) (Figure 2I).

### 3.4 Comparative and subgroup analyses

Open vs. Minimally Invasive Surgery: A comparison between the 30 open surgery cases and the 6 minimally invasive surgery (MIS) cases revealed that the MIS group had a non-significant trend towards lower complication rates (16.7% vs. 13.3%) and shorter average length of hospital stay (4.8 days vs. 12.5 days), though the small sample size in the MIS group limits statistical power.

Obese vs. Non-Obese Patients: Subgroup analysis of the 17 obese patients showed they were more likely to have a mesh placed during repair (65% vs. 39% in non-obese patients) and had a higher incidence of postoperative adverse events (23.5% vs. 4.3% in non-obese patients), reinforcing obesity as a significant risk factor.

### 3.5 Postoperative complications

Most patients (35/40, 88%) had no significant complications. Reported complications included: Pneumothorax: 1 case, Subcutaneous hematoma: 1 case, Surgical site infection: 1 case, Recurrence: 1 case, Mortality: 1 case (Figure 2J).

### 3.6 Prognostic factors for poor outcomes

Univariate analysis identified three significant risk factors for composite adverse events (death/recurrence/major complications): Obesity (HR 2.37, 95% CI 1.42–3.96; *P* = 0.001), Defect size >5 cm (HR 2.89, 95% CI 1.73–4.83; *P* < 0.001) and Non-mesh repair (HR 1.98, 95% CI 1.21–3.24; *P* = 0.006). In multivariate analysis, only obesity (adjusted HR 2.05, 95% CI 1.18–3.56; *P* = 0.011) and large defect size (>5 cm: aHR 2.41, 95% CI 1.39–4.18; *P* = 0.002) remained independent predictors (Table 1). In multivariate analysis, non-use of mesh was associated with a 62% increased risk of adverse outcomes (aHR 1.62, 95% CI 0.95–2.76, *p* = 0.077), suggesting a non-significant protective trend of mesh reinforcement.

**Table 1:** Univariate and multivariate analysis of adverse outcomes.

1. Univariate Analysis		
Variable	HR (95% CI)	*p*-value
Age ≥60 years	1.25 (0.82–1.91)	0.297
Male sex	1.08 (0.65–1.80)	0.769
Obesity	2.37 (1.42–3.96)	0.001
Defect >5cm	2.89 (1.73–4.83)	<0.001
Open surgery	1.52 (0.93–2.49)	0.094
Mesh use	0.51 (0.31–0.83)	0.006
2. Multivariate Analysis		
Variable	aHR (95% CI)	*p*-value
Obesity	2.05 (1.18–3.56)	0.011
Defect >5cm	2.41 (1.39–4.18)	0.002
Mesh use	0.62 (0.36–1.05)	0.077

HR: Hazard ratio, aHR: adjusted hazard ratio, CI:confidence interval, *P*-values in bold were statistically significant.

## 4 Discussion

Transdiaphragmatic intercostal hernia (TDIH) is a rare clinical condition that often occurs secondary to traumatic diaphragmatic injury and is frequently overlooked or misdiagnosed. This study reviewed one TDIH case treated at our institution and 39 cases reported in the literature, summarizing the clinical characteristics, diagnosis, treatment methods, and postoperative management of TDIH, providing new perspectives for better understanding and managing this condition.

Our findings are largely consistent with previous smaller series. The male predominance (75%) and left-sided preponderance (58%) we observed align with the literature, likely due to higher rates of trauma in males and the protective effect of the liver on the right hemidiaphragm.

The pathogenesis of TDIH is typically associated with blunt trauma, penetrating trauma, or sudden changes in intra-abdominal pressure (1). The classic mechanism involves diaphragmatic rupture allowing abdominal contents to protrude through the defect into the thoracic cavity, with some cases showing further herniation through the intercostal space (2). Trauma and respiratory diseases are common predisposing factors, while spontaneous and iatrogenic causes are rare. In our reviewed cases, some patients initially presented with mild and nonspecific symptoms, making early diagnosis particularly challenging. Therefore, comprehensive history-taking is crucial for diagnosis, especially in patients with suspected TDIH who have a history of trauma or severe coughing.

Among the 40 cases, the mean patient age was 62.5 ± 13.7 years, with 55% (22 cases) aged 60 years or older. This age distribution aligns with the trend of TDIH being more common in elderly patients (29), possibly due to age-related degenerative changes in the diaphragm and increased abdominal pressure. Male patients accounted for 75% (30 cases) vs. 25% (10 cases) female patients, consistent with most relevant studies (29). Left-sided TDIH was more frequent than right-sided, likely due to the protective effect of the liver on the right side (38).

Common clinical symptoms included dyspnea, abdominal pain, chest pain, cough, and palpable masses. Atypical symptoms included swelling sensation, nausea/vomiting, intestinal obstruction, and hematoma/bruising. Notably, despite varied clinical presentations, some patients had mild and nonspecific symptoms, potentially leading to missed or incorrect diagnoses, especially when symptoms were atypical or medical history was incomplete. Therefore, clinicians should maintain high suspicion for TDIH, particularly in patients with trauma history, chronic cough, or other risk factors. Our risk analysis reveals that “obesity” and “large diaphragmatic defects” independently predict adverse outcomes in TDIH patients. Potential mechanisms include: metabolic syndrome-induced diaphragmatic dysfunction, and increased biomechanical tension at the repair interface.

Strategies to Reduce Recurrence: Based on our findings, we recommend a low threshold for mesh reinforcement in patients with defect sizes >5 cm or in those with obesity to reduce recurrence risk. Furthermore, the choice of surgical approach should be individualized; while open surgery remains a robust option, minimally invasive techniques, including robotic-assisted surgery, offer enhanced visualization in the confined thoracic space and may facilitate precise mesh placement in complex cases, potentially improving outcomes.

Imaging examinations are crucial for TDIH diagnosis. CT is considered the gold standard (4, 14), clearly demonstrating diaphragmatic defects and herniated contents. Ultrasound also has diagnostic value as an adjunct tool, especially in thin patients or those with typical symptoms (37). However, variations in patient body habitus, herniated contents, and defect characteristics may lead to diagnostic challenges, particularly in obese patients. Therefore, integrating clinical symptoms, history, and imaging findings is essential for accurate diagnosis.

Surgical repair remains the mainstay of TDIH treatment (5). In our study, 75% of patients (30 cases) underwent open surgery. The timing and approach should be individualized based on symptoms, hernia type, and severity of diaphragmatic injury, primarily considering the size of herniated contents, extent of diaphragmatic defect, and patient's overall condition. Open repair is typically preferred for acute cases, effectively addressing both diaphragmatic and intercostal defects. Selected cases with mild symptoms or delayed presentation may be managed non-operatively (27), particularly in elderly or frail patients.

Tension-free mesh repair has become increasingly popular, as it reduces recurrence risk and avoids chronic pain from permanent sutures (5). Mesh use should be determined intraoperatively. Our case employed absorbable interrupted sutures for diaphragmatic repair without significant tension. Although mesh reinforcement improves outcomes, complications including adhesions, mesh rejection, and recurrence persist (5). Our study documented one recurrence case, with literature reporting approximately 28.6% recurrence rate (4), particularly higher in cases with severe trauma or incomplete tension relief during repair. Therefore, postoperative follow-up is critical for early recurrence detection and potential reoperation. While no standardized surgical approach exists, minimally invasive techniques (e.g., laparoscopy and robotic-assisted surgery) have emerged as preferred options (5).

Postoperative management is equally important, particularly for preventing complications and recurrence. Varying recurrence rates across studies suggest associations with surgical techniques, postoperative care, and patient factors. Close monitoring of respiratory function and abdominal pressure changes helps reduce complications.

### 4.1 Clinical implications and future directions

Our study consolidates the clinical profile of TDIH and provides evidence for risk stratification. To guide clinical practice, we propose a straightforward management principle: high clinical suspicion in at-risk individuals → confirmatory CT scan → individualized surgical planning with strong consideration for mesh repair in high-risk patients (obesity, defect >5 cm). Future efforts should focus on prospective, multi-center registries to validate these risk factors and assess long-term outcomes of novel minimally invasive and robotic techniques. Study limitations include relatively small sample size and reliance on institutional and literature cases. Future studies should expand sample sizes to validate our findings. In conclusion, TDIH is a rare but clinically significant condition where timely diagnosis and appropriate treatment are crucial for prognosis. Future research should focus on developing more accurate imaging techniques and personalized treatment strategies to improve patient outcomes and reduce recurrence risk.

## Data Availability

The original contributions presented in the study are included in the article/Supplementary Material, further inquiries can be directed to the corresponding author.
